# Custom Loss Functions in XGBoost Algorithm for Enhanced Critical Error Mitigation in Drill-Wear Analysis of Melamine-Faced Chipboard

**DOI:** 10.3390/s24041092

**Published:** 2024-02-07

**Authors:** Michał Bukowski, Jarosław Kurek, Bartosz Świderski, Albina Jegorowa

**Affiliations:** 1 Institute of Information Technology, Warsaw University of Life Sciences, 02-776 Warsaw, Poland; michal.bukowski@buksoft.pl (M.B.); bartosz_swiderski@sggw.edu.pl (B.Ś.); 2 Institute of Wood Sciences and Furniture, Warsaw University of Life Sciences, 02-787 Warsaw, Poland; albina_jegorowa@sggw.edu.pl

**Keywords:** drill-wear analysis in woodworking, XGBoost custom loss functions, melamine-faced chipboard machining, critical error reduction in classification

## Abstract

The advancement of machine learning in industrial applications has necessitated the development of tailored solutions to address specific challenges, particularly in multi-class classification tasks. This study delves into the customization of loss functions within the eXtreme Gradient Boosting (XGBoost) algorithm, which is a critical step in enhancing the algorithm’s performance for specific applications. Our research is motivated by the need for precision and efficiency in the industrial domain, where the implications of misclassification can be substantial. We focus on the drill-wear analysis of melamine-faced chipboard, a common material in furniture production, to demonstrate the impact of custom loss functions. The paper explores several variants of Weighted Softmax Loss Functions, including Edge Penalty and Adaptive Weighted Softmax Loss, to address the challenges of class imbalance and the heightened importance of accurately classifying edge classes. Our findings reveal that these custom loss functions significantly reduce critical errors in classification without compromising the overall accuracy of the model. This research not only contributes to the field of industrial machine learning by providing a nuanced approach to loss function customization but also underscores the importance of context-specific adaptations in machine learning algorithms. The results showcase the potential of tailored loss functions in balancing precision and efficiency, ensuring reliable and effective machine learning solutions in industrial settings.

## 1. Introduction

Machine learning (ML) has become a cornerstone in various industrial applications, revolutionizing how data are utilized to make critical decisions. Among the plethora of ML algorithms, eXtreme Gradient Boosting (XGBoost) has emerged as a powerful tool, particularly for classification tasks. However, the conventional implementation of XGBoost often falls short in complex multi-class classification scenarios, where the stakes of misclassification are high. This paper addresses the pivotal role of customizing loss functions within the XGBoost framework to cater to the nuanced demands of specific industrial applications, particularly focusing on the drill-wear analysis in melamine-faced chipboard production—a critical process in the furniture manufacturing industry [1,2,3,4,5].

Laminated chipboard panels are commonly applied materials in the furniture industry, known for their cost-effectiveness. However, they also present unique challenges during the production process, such as wear and tear on tools and unpredictability in material consistency due to variations in glue density and the presence of air pockets [6,7,8,9]. Consequently, accurately determining the precise timing for tool replacement during drilling operations is a critical issue that calls for automated solutions [10,11,12].

Tool condition monitoring in wood-based industries, including furniture manufacturing, has been extensively explored, with various methodologies already in place. These include using a range of sensors to measure parameters such as acoustic emission, noise, vibrations, cutting torque, and feed force [13,14,15,16,17]. Furthermore, the application of ML algorithms in this domain has been diverse, ranging from monitoring overall machining processes to identifying different wood species [18,19,20].

Recent trends have shifted towards leveraging simplified inputs, such as images, with transfer and deep learning methodologies that have been proven effective [21,22]. These approaches are further enhanced by data augmentation and classifier ensemble techniques [23,24]. The choice of classifiers also significantly impacts solution quality [25,26,27,28].

At the heart of ML-based solutions is the crucial step of feature extraction. In the context of drill wear recognition, numerous parameters derived from images of drilled holes can be considered. There is a growing need for systematic methodologies to select the most compelling feature extraction methods, highlighting the potential benefits of different approaches [10,11,13,17,21,22,23,24,26,27,28,29,30,31,32].

In recent years, the eXtreme Gradient Boosting (XGBoost) [33,34,35,36,37] algorithm has emerged as a powerful tool for tackling complex predictive tasks. Its ability to handle non-linear relationships and interactions between features makes it particularly suitable for industrial applications where precision and reliability are paramount. However, traditional applications of XGBoost may not fully address the nuances of critical error mitigation in the context of drill-wear analysis.

This study introduces novel custom loss functions within the XGBoost framework, aiming to enhance the model’s performance in identifying and mitigating critical errors in the drill-wear analysis of melamine-faced chipboard. The rationale behind customizing loss functions is to fine-tune the algorithm’s sensitivity to errors that have significant implications for production quality and efficiency. By tailoring the loss functions, we aim to achieve a more nuanced and contextually aware model that can provide more accurate predictions and insights, thereby reducing downtime and improving overall manufacturing processes.

Furthermore, the adaptation of XGBoost with these custom loss functions presents a novel approach in the field of industrial machine learning applications. It exemplifies the potential for algorithmic customization to meet specific industrial challenges, paving the way for more targeted and effective machine learning solutions in various sectors. The following sections detail the methodology adopted for this study, the development and implementation of the custom loss functions, and an analysis of the results obtained from applying this novel approach to drill-wear analysis in melamine-faced chipboard processing.

Despite the advancements in applying ML algorithms in the wood industry, a significant research gap remains in addressing the specific challenges of drill-wear analysis in the production of melamine-faced chipboards. The current study aims to fill this gap by exploring the use of custom loss functions in the XGBoost algorithm for enhanced critical error mitigation. The motivation behind this research stems from the industry’s need for precise tool condition monitoring, which directly impacts production quality and efficiency.

A critical aspect that differentiates this study from conventional approaches is the specific goal set by industry experts. In this context, the primary objective is not merely to achieve the highest accuracy metric but to minimize the number of critical errors. This means that the model should ideally avoid misclassifying the extreme classes, specifically minimizing errors between the ‘Green’ and ‘Red’ classes, even if it leads to a higher overall accuracy error. The rationale is that in industrial applications, particularly in the context of drill-wear analysis, the consequences of misclassifying a tool in need of immediate replacement (Red) as in good condition (Green), or vice versa, can be far more detrimental than a lower accuracy in less critical classifications. This approach represents a significant shift from traditional ML objectives and underscores the importance of tailoring ML solutions to meet specific industrial needs. The pursuit of this goal forms the core of our research, aiming to develop a model that aligns with the practical demands of the furniture manufacturing industry, ensuring reliability and effectiveness in a real-world production environment.

Similarly to the exploration of various custom loss functions for different learning requirements in this article, future work could also delve into the analysis of network sensitivity, as demonstrated in several key studies. Naik and Kiran (2021) proposed a novel sensitivity-based method for feature selection within deep neural networks, showing its efficacy in identifying important features across datasets. Asheghi et al. (2020) enhanced neural network models for sediment load prediction by employing diverse sensitivity analysis methods, leading to improved model predictability. Yeung et al. (2010) discussed the significance of sensitivity analysis in artificial neural networks for engineering system design in their comprehensive book. Abbaszadeh Shahri et al. (2022) introduced an innovative approach for uncertainty quantification in groundwater table modeling, leveraging automated predictive deep learning for more accurate predictions. Ghaderi and Abbaszadeh Shahri (2022) developed a hybrid intelligence model to delineate soil layers using clay sensitivity, showcasing the potential of hybrid models in geo-engineering applications. These studies collectively highlight the crucial role of sensitivity analysis in enhancing model performance and reliability across various domains [38,39,40,41,42].

## 2. Materials and Methods

### 2.1. Data Collection

**Figure 1:**
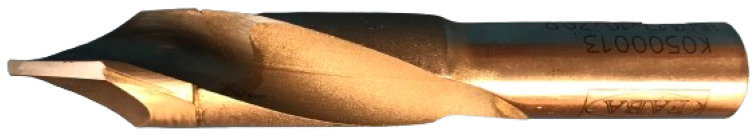
The FABA WP-01 drill used during experiments.

**Figure 2:**
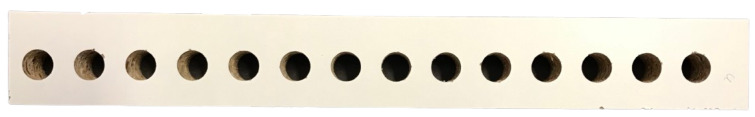
Example initial profile scans with drilled holes.

For the experimental analysis, image data were acquired in a setting that closely resembled a real-world production environment. The drilling process utilized a sophisticated automated CNC workstation, specifically the Busselato Jet 100 model, manufactured in Piovenne Rochette, Italy. Drilling was executed using a standard FABA drill with a diameter of 12 mm (model WP-01 (FABA, Treviso, Italy), see Figure 1). Operational parameters, including a rotation speed of 4500 RPM and a feed rate of 1.35 m/min, were selected based on the recommendations provided by the drill manufacturer. The material used for drilling was standard laminated chipboard, specifically the KRONOPOL U 511 model (Kronopol, Żary, Poland), featuring a thickness of 18 mm, a standard specification in the furniture industry [43,44,45]. The experiment involved the preparation of 610 distinct chipboard profiles from the laminated chipboard panels [46] (illustrated in Figure 2). Each profile measured 300 mm  × 35 mm  × 18 mm and included 14 evenly spaced holes located centrally. This arrangement was chosen to minimize the impact of material stress on the quality of the holes, thus ensuring a consistent and uniform drilling area across the panel. The total number of holes drilled in all the panels amounted to 8540. Regular inspections were conducted using a Mitutoyo microscope (model TM-500, based in Kawasaki, Japan) to assess the condition of the drill throughout the process. According to the observed wear, the drill was categorized into one of three distinct classes. The first, Good (Green), is indicative of a new drill in optimal condition for use. The second, Worn (Yellow), signifies a drill in an exemplary state; it is still functional but potentially needs replacing soon. The third, Requiring Replacement (Red), represents a drill that is no longer usable and needs immediate replacement.

In the evaluation of drill conditions during inspections, the state of the drill was determined based on a wear parameter, denoted as *W*. This parameter represents the disparity in the width of the cutting edge when comparing a new drill (measurement taken near the outer corner) to the width of the same section in the tool under review. The unit of measurement for *W* is millimeters [mm]. Based on the guidelines provided by the manufacturer, the condition of the drill is classified into distinct categories. A drill is considered in the ‘Good’ condition if *W* is less than 0.2 [mm]. It falls under the ‘Worn’ category if *W* lies between 0.2 [mm] and 0.35 [mm]. When *W* exceeds 0.35 [mm], the drill is deemed to require replacement.

Subsequent to these assessments, the obtained profiles underwent scanning as part of the further analysis processes. The scanning was conducted at a resolution of 1200 dots per inch [dpi]. For examples of sample profiles that exhibit holes drilled by tools of varying wear conditions, refer to Figure 2.

A total of 610 images of chipboard profiles were captured and subsequently segregated into distinct files corresponding to each hole. This organization facilitated not only the individual examination of each case but also the automation of subsequent image processing stages. The composition of the dataset applied is detailed in Table 1.

### 2.2. Feature Extraction Techniques

**Figure 3:**
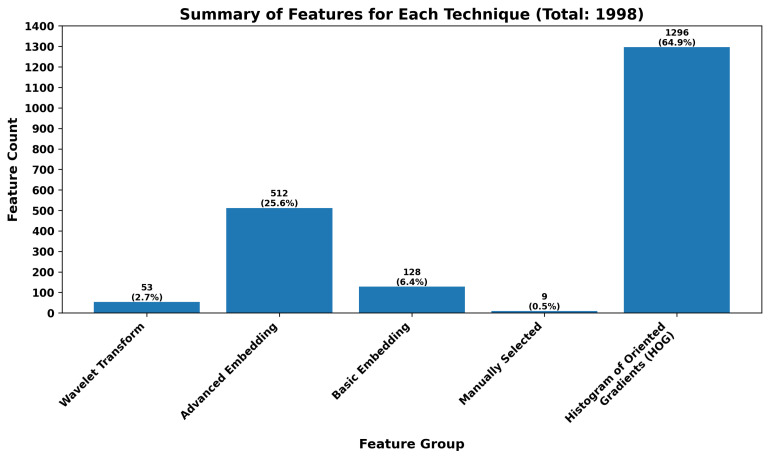
Summary of features for each technique.

Several methods for generating features, resulting in five primary feature groups, have been selected. The figure below (Figure 3) summarizes the total count of features derived from each method.

The bar chart presented provides a comprehensive summary of features utilized across different feature extraction techniques. A total of 1998 features have been cataloged, with their distribution across various methods explicitly detailed.

At the lower end of the spectrum, Wavelet Transform accounts for a modest portion, representing 53 features, which is approximately 2.7% of the total. High-level Embedding (based on Pretrained Convolutional Neural Network-CNN-512 variables) shows a more significant contribution with 512 features, forming 25.6% of the overall count. Low-level embedding (based on Pretrained Convolutional Neural Network-CNN-128 variables) makes up 6.4%, while Manual Selection is the least represented with only nine features, equating to a mere 0.5%.

The most substantial proportion of features is attributed to the Histogram of Oriented Gradients (HOG) technique, which encompasses a staggering 1296 features. This figure constitutes 64.9% of the total feature count, underscoring the predominant reliance on this method in the feature extraction process.

The chart effectively illustrates the reliance on different feature extraction techniques in the analysis, with HOG standing out as the most utilized method by a significant margin.

#### 2.2.1. 2-D Morlet Wavelets in Wavelet Scattering Image Decomposition

The wavelet scattering approach, often referred to as the scattering transform or wavelet transform, applies convolutions of input signals to gauge similarities among objects. This approach’s efficiency in recognizing local correlations arises from the merging of two akin values.

Tracing the origins of wavelet scattering brings us to the Fourier transform’s development, an essential technique in signal processing. However, Fourier representation struggles with instability at higher frequencies during signal deformation. This is attributed to the sine wave’s limitations in localizing frequency-specific information [47].

Addressing this issue, the wavelet transform decomposes [48] a signal into a set of varying wavelets, effectively pinpointing the signal’s high-frequency elements. The translation property inherent in the wavelet operator ensures covariance in the representation—a shift in the signal correspondingly shifts its wavelet coefficients, posing a challenge for comparing signals with translations. Achieving translation invariance is crucial for applications such as classification.

Fundamentally, the wavelet transform operates on the dot product using a kernel. This kernel is a wavelet function tailored to meet time-frequency analysis needs. The wavelet’s frequency domain spectrum is adjustable through the scale parameter *a*, while the time domain spectrum is adjusted via the offset parameter *b*.

The applied decomposition utilizes a Morlet wavelet, incorporating both real and complex components, and is graphically represented in the time domain [49,50]:

(1) $$\Psi_{\sigma }\left(t\right)=c_{\sigma }\pi^{-\frac{1}{4}}e^{-\frac{1}{2}t^{2}}\left(e^{i\sigma t}-\kappa_{\sigma }\right)$$

where $\kappa_{\sigma }$ serves as the admissibility criterion:

(2) $$\kappa_{\sigma }=e^{-\frac{1}{2}\sigma^{2}}$$

and $c_{\sigma }$ is the normalization constant:

(3) $$c_{\sigma }={\left(1+e^{-\sigma^{2}}-2e^{-\frac{3}{4}\sigma^{2}}\right)}^{-\frac{1}{2}}$$

The fast Fourier transform for this system is expressed as:

(4) $${\hat{\Psi }}_{\sigma }\left(\omega \right)=c_{\sigma }\pi^{-\frac{1}{4}}\left(e^{-\frac{1}{2}{\left(\sigma -\omega \right)}^{2}}-\kappa_{\sigma }e^{-\frac{1}{2}\omega^{2}}\right)$$

This process involves a Gaussian-windowed sine wave conducting the convolution across various frequency locations using indexed wavelets ($\Psi_{\nu }$), with the wavelet transform ($\Psi_{x}$) producing a range of dispersion coefficients.

This methodology, when applied to the chosen dataset, yielded a total of 53 distinct features.

The implementation of wavelet feature extraction from images, as described in the context of 2-D Morlet wavelets and wavelet scattering image decomposition, is detailed in Algorithm 1, where the specific steps of the procedure are outlined.  
**Algorithm 1** Wavelet Feature Extraction from Images1:**procedure** WaveletFeatureExtraction2:    Define root folders and classes for image datasets        ▹ for 5 drills and 3 classes3:    Create imageDatastore instances for each dataset and classes4:    **for** each dataset combination (1 through 5) **do**5:        Combine training sets from other four datasets6:        Assign corresponding labels to the combined training set7:        Set aside one dataset as the testing set8:        Extract wavelet features for both training and testing sets9:        Assign labels to the training and testing sets10:    **end for**11:    Save all wavelet features and labels to a file12:**end procedure**13:                                      ▹14:**procedure** getWaveletScattering2(ImageDatastore)15:    Read all images from the datastore16:    Define wavelet scattering for ImageSize=[224 224]17:    Initialize empty array for all features18:    **for** each image in the datastore **do**19:        Extract wavelet features using defined wavelet scattering20:        Compute the mean of the features21:        Append the features to the features array22:    **end for**23:    **return** features array24:**end procedure**

#### 2.2.2. Pretrained Network: ResNet-18 for Extracting Lower-Level and High-Level Features

ResNet-18, part of the Residual Network family, is a convolutional neural network (CNN) that has been pretrained on more than a million images from the ImageNet database [51,52]. Despite its overall depth, the ‘18’ in ResNet-18 signifies the count of its primary layers: the convolutional and fully-connected layers [53]. The network is 18 layers deep and can classify images into 1000 object categories, such as keyboard, mouse, pencil, and many animals. As a result, the network has learned rich feature representations for a wide range of images.

The network layers include a series of convolutional layers, each followed by batch normalization and a rectified linear unit (ReLU) activation function. The defining characteristic of ResNet is the introduction of skip connections (or shortcut connections) that bypass one or more layers. These connections are represented as identity mappings, adding the output from an earlier layer to a later layer. This helps to mitigate the vanishing gradient problem that affects deep networks, allowing for the training of much deeper networks.

In our work, we utilize ResNet-18 as a feature extractor. The extracted features from the intermediate layers of the network are used as input to machine learning models for various classification tasks. The pretrained network is beneficial in scenarios where the dataset is not sufficiently large to train a deep network from scratch. By leveraging transfer learning, we can apply the powerful feature representations learned by ResNet-18 on ImageNet to new tasks with limited data.

**Figure 4:**
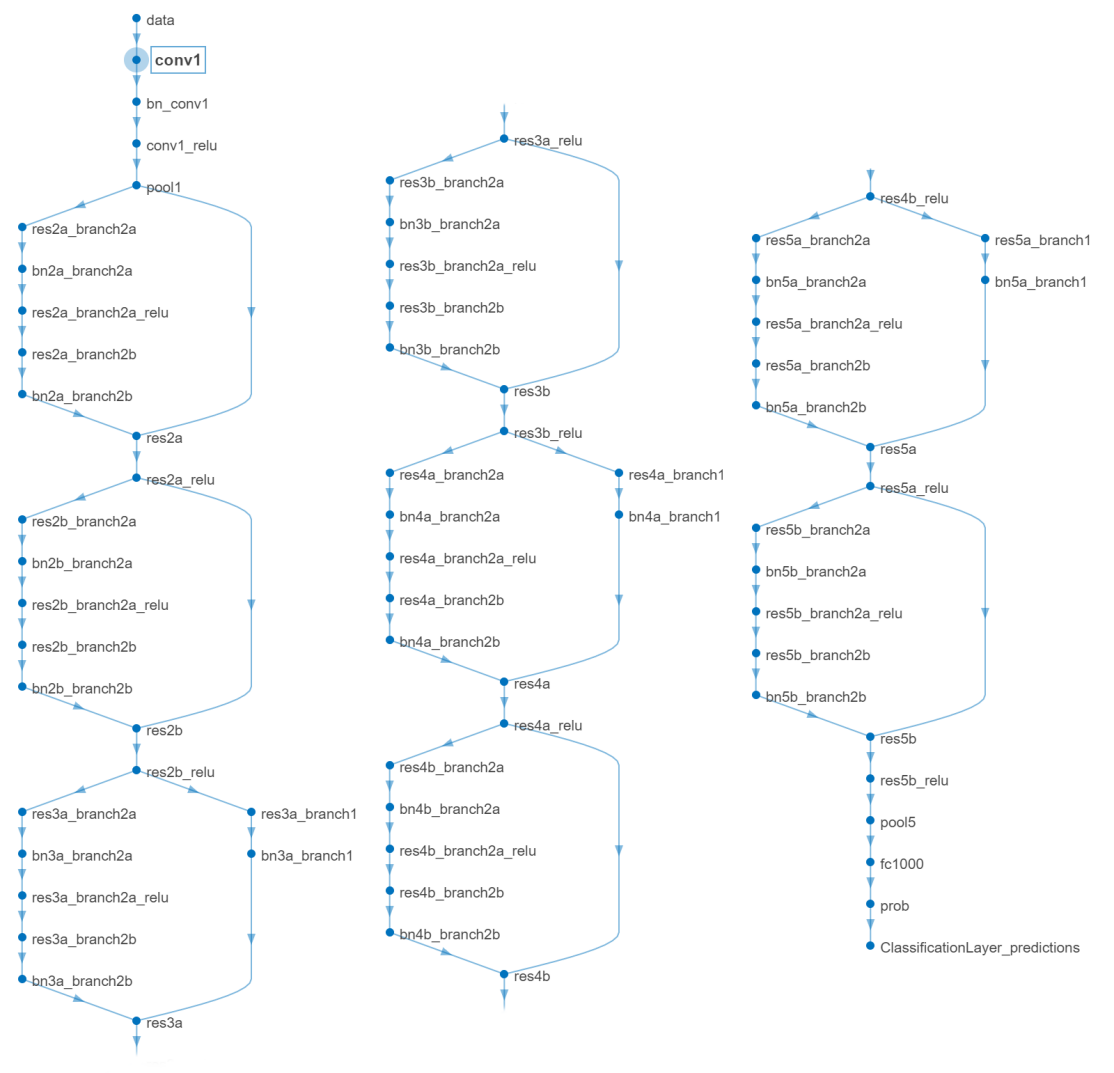
The architecture of the pretrained ResNet-18 network.

The architecture of ResNet-18, as depicted in Figure 4, consists of an initial convolutional layer followed by a series of residual blocks that make up the core of the network. These residual blocks contain the skip connections that are the hallmark of the ResNet architecture. The network concludes with a global average pooling layer and a fully connected layer that outputs the class probabilities.

For a comprehensive understanding of ResNet-18 architecture, including the specific functions of each layer, Table 2 has been added. This table provides a detailed layer-by-layer description of the ResNet-18 network, highlighting the critical roles of layers 35 and 68 in feature extraction.

#### 2.2.3. High-Level Features Feature Extraction Using Pretrained Convolutional Networks

In our study, high-level feature extraction was conducted using features from the network’s 68th layer called ‘pool5’. This layer was selected for its comprehensive accumulation of image-derived features essential for classification. Specifically, 512 distinct features were extracted for each image. The network’s pre-training on the expansive ImageNet [51] database eliminates the need for additional training for effective feature extraction. For a complete layer-by-layer breakdown of the ResNet-18 architecture, and specifically the significance of the 68th layer in feature extraction, refer to Table 2.

The process of high-level feature extraction utilizing a pretrained convolutional neural network (CNN), such as ResNet-18, is summarized in Algorithm 2. This algorithm outlines the key steps for preprocessing the images and extracting features from a specific layer of the CNN.
**Algorithm 2** CNN Feature Extraction Using ResNet-18’s 35th/68th Layer1:**procedure** CNNFeatureExtraction2:    Initialize root folders for each dataset         ▹ for 5 drills3:    Define categories: Green, Yellow, Red      ▹ for three subfolders4:    Create imageDatastore instances for each dataset5:    Combine datasets for training and testing6:    Load pretrained ResNet-18 model7:    Define feature extraction layer (e.g., 68th layer or 35th layer)8:    **for** each combined dataset (1 through 5) **do**9:        Read image10:        Preprocess image according to CNN input requirements11:        Extract features using the specified CNN layer12:        Store extracted features13:    **end for**14:    Output the set of extracted features15:**end procedure**

#### 2.2.4. Low-Level Extracting of Features Using ResNet-18’s 35th Layer

This study also investigates the efficacy of initial-stage features in addressing the problem at hand. To this end, a feature set was derived from the 35th layer of the ResNet-18 architecture. This process mirrored the methodology used for extracting features from the network’s 68th layer. Typically, the initial layers of a deep learning network such as ResNet-18 are known for capturing more basic, rudimentary features. These layers generally maintain a higher spatial resolution and result in a greater total count of activations. Notably, the 35th layer is pivotal as it is the final layer contributing 128 novel features, ensuring an optimal spatial representation at a resolution of 28 × 28. A detailed description of the ResNet-18 network architecture, including the specific role of the 35th layer, can be found in Table 2.

The process of low-level extracting of features using the 35th layer of the ResNet-18 architecture is detailed in Algorithm 2. This approach leverages the initial-stage features from the CNN for our specific classification task.

#### 2.2.5. Manually Defined Feature Set

The extraction of features included consideration of physical properties derived from analyzing photographs of the defects. Specifically in furniture production, different types of flaws impact the quality of the end product differently. For example, a hole with a single, extensive chip is more detrimental than one with multiple, minor chips near the perimeter. The latter can be concealed during the assembly of the furniture, whereas the former often leads to the rejection of the product. To encapsulate these distinctions, a set of manually defined features was developed, encompassing nine key attributes:

1. Diameter of the smallest circle encompassing the hole;
2. Diameter of the largest circle that can fit inside the hole;
3. Variation in hole diameters;
4. Total area covered by holes;
5. Area of the convex hull;
6. Total perimeter length;
7. Length of the longest axis of the ellipse fitting the image;
8. Length of the shortest axis of the ellipse fitting the image;
9. Solidity (ratio of area to convex hull area).

The specific steps for generating these features are detailed in Algorithm 3.
**Algorithm 3** Generation of Individual Image Features Using Custom Method**procedure** GenerateManualFeatures (file)    Read image *I* from file    Adjust *I* and convert to binary images bw    Label connected components in bw to *L*    Extract properties of regions in *L* into *s*    Find the largest region in *s* as circle    Extract edge coordinates *x*, *y* from circle    Calculate inscribed circle properties: *R*, cx, cy    Calculate convex hull points *x*, *y* of circle    Calculate circumscribed circle properties: center, radius    Compute features: *R*, radius, radius−R, s(numer).Area, etc.    **return** computed features as ManualFeatures**end procedure**

#### 2.2.6. Histogram of Oriented Gradients (HOG) for Feature Extraction

Histogram of Oriented Gradients (HOG) is a type of feature descriptor, similar to SIFT (scale-invariant feature transform) and SURF (speeded-up robust feature), widely used in the realm of computer vision for extracting features [54,55,56,57,58]. HOG is particularly adept at capturing an object’s form or structural characteristics. Unlike basic edge detection, which distinguishes edge pixels, HOG takes this a step further by considering the directionality of edges, which is achieved by analyzing the gradient and orientation of the edges.

During the process of analysis, the image is segmented into smaller sections. For each of these segments, the gradients and their orientations are calculated, leading to the generation of individual histograms for each segment. These histograms are formulated on the basis of the gradients and the orientations of pixel values within the segment.

The overall procedure encompasses three phases: initial data preparation, calculation of gradients, and the ascertainment of orientations derived from the gradient data. Each alteration is assessed in both dimensions of a 2-D object, i.e., along the X and Y axes. The variations between these two dimensions form the foundation of the traditional Pythagorean methodology used in calculating gradient magnitude. Furthermore, the angle ϕ, indicating direction, is ascertained using these calculated metrics.

Application of the HOG method has enabled the extraction of 1296 distinct features from smaller regions, each with a defined cell size of 32 × 32.

The process of extracting HOG features is detailed in Algorithm 4.
**Algorithm 4** Extract HOG Features**procedure** extractHOGFeatures (image)    gradients← computeGradients(image)    cells← divideIntoCells(image)    hogFeatures←[]    **for** each cell in cells **do**        histogram← initializeHistogram        **for** each pixel in cell **do**           gradient←gradients[pixel]           bin← findOrientationBin(gradient)           addToHistogram(histogram, bin, gradient.magnitude)        **end for**        normalizedHistogram← normalizeHistogram(histogram)        appendToFeatureVector(hogFeatures,normalizedHistogram)    **end for**    **return** hogFeatures**end procedure**

### 2.3. Extreme Gradient Boosting—XGBoost

The eXtreme Gradient Boosting (XGBoost) algorithm is a highly efficient and scalable implementation of Gradient Boosting machines, a type of machine learning algorithm for regression and classification problems. XGBoost was developed by Chen and Guestrin (2016) [59] and has gained popularity in machine learning competitions due to its performance and speed.

Gradient Boosting is a machine learning technique for classification problems, which produces a prediction model in the form of an ensemble of weak prediction models, typically decision trees. The method was further refined by Friedman (2000, 2001, 2002) [60,61,62] through the introduction of techniques such as stochastic Gradient Boosting and improvements in loss function optimization.

XGBoost is a refined and enhanced version of Gradient Boosting [63]. The core of the XGBoost algorithm involves sequentially adding predictors to an ensemble, each one correcting its predecessor. However, unlike traditional Gradient Boosting, XGBoost utilizes a more regularized model formalization to control overfitting, which gives it better performance.

### 2.4. Key Features of XGBoost

The key features of XGBoost include:

- Regularization: This includes L1 (Lasso Regression) and L2 (Ridge Regression) regularization, which helps in reducing overfitting.
- Handling Sparse Data: XGBoost is designed to handle sparse data from the ground up.
- Tree Pruning: XGBoost uses a depth-first approach for tree pruning, unlike the level-wise approach, making it more efficient.
- Handling Missing Values: XGBoost has an in-built routine to handle missing values.
- System Optimization: The system is optimized for distributed computing and can handle large datasets efficiently.

XGBoost was selected for its numerous benefits over the original model. It integrates regularization principles into the training process and supports parallel computation. The model also comes equipped with capabilities to address missing data and includes built-in procedures for cross-validation and tree pruning. Collectively, these features enable XGBoost potentially to outperform its predecessors [64].

In our analysis, eXtreme Gradient Boosting was configured with the following parameters:

- Number of Boosting Stages (M) = 50;
- Loss Function = ‘log-loss’;
- Maximum Depth of Trees = 3;
- Learning Rate = 0.1;
- Minimum Samples per Leaf = 1.

### 2.5. Loss Functions

Loss functions play a main role in the realm of machine learning (ML) modeling. They are the guiding metrics that an AI model aims to minimize during the training process. The choice of a loss function significantly influences the behavior and performance of the model, as it dictates how the model’s predictions are evaluated against the actual outcomes.

The primary purpose of loss functions in AI models is to quantify the difference between the predicted values and the actual values. During the training phase, AI models use algorithms to adjust their parameters. The direction and magnitude of these adjustments are determined based on the loss function. A well-chosen loss function ensures that the model learns the correct patterns and makes accurate predictions.

While standard loss functions for classification tasks are practical in many scenarios, they are not one-size-fits-all solutions [65,66,67]. In particular, these functions may not perform optimally in situations with imbalanced data or when certain types of errors have more severe consequences than others.

In multi-class classification tasks, edge classes often represent categories with fewer samples or higher misclassification costs. Standard loss functions may not adequately penalize errors in these edge classes, leading to suboptimal model performance for these critical categories:

- Addressing Class Imbalance: Loss function modification can help address the class imbalance by assigning higher penalties for errors in underrepresented classes. This ensures that the model does not overlook these classes during the learning process.
- Focusing on Critical Errors: In many real-world applications, certain misclassifications are more costly than others. Customized loss functions can be designed to impose heavier penalties for specific types of critical errors, thereby reducing their occurrence.
- Improving Model Sensitivity: Modifying loss functions can improve the model’s sensitivity towards edge classes, enhancing its ability to detect and correctly classify instances belonging to these classes.

Loss functions are more than just a measure of a machine learning model’s error. They are instrumental in shaping the learning process. Customizing loss functions, particularly in the context of multi-class classification, can lead to significant improvements in model performance, especially for edge classes. This customization enables the development of AI models that are not only accurate but also aligned with the specific needs and priorities of their application domains.

### 2.6. XGBoost’s Default Loss Function for Multi-Class Classification

XGBoost, a popular machine learning algorithm for boosted trees, uses different loss functions depending on the nature of the problem. For multi-class classification tasks, where the objective is to categorize instances into one of three or more classes, XGBoost defaults to the ‘multi:softprob’ loss function.

The ‘multi:softprob’ function is designed for multi-class classification problems. It calculates the probability of each class for an instance and uses these probabilities to minimize the loss during the training process.

The loss for a single instance is given by the negative log-likelihood of the true class [68]:

(5) $$L\left(y,\hat{y}\right)=-\sum_{k=1}^{K}y_{k}\log\left({\hat{y}}_{k}\right)$$

Here, *K* represents the total number of classes, $y_{k}$ is a binary indicator (0 or 1) if class label *k* is the correct classification for the observation, and ${\hat{y}}_{k}$ is the predicted probability of the observation being of class *k*.

#### Pseudocode for ‘multi:softprob’

The following pseudocode (Algorithm 5) outlines the basic approach of the ‘multi:softprob’ function during the training phase of XGBoost.
**Algorithm 5** Pseudocode for multi:softprob in XGBoost**for** each iteration **do**    **for** each training instance *i* **do**        Calculate predictions ${\hat{y}}_{i}$ for all classes        Compute probabilities using the softmax function        **for** each class *k* **do**           Calculate $L(y_{i},{\hat{y}}_{i})$ for class *k*        **end for**        Update the model to minimize the loss    **end for****end for**

This pseudocode simplifies the complex process behind XGBoost’s ‘multi:softprob’ function while still capturing the essence of predicting class probabilities and updating the model to minimize loss.

### 2.7. Weighted Softmax Loss Variant 1

The ‘WeightedSoftmaxLossVariant1’ function extends the traditional Softmax Loss Function by introducing class-specific weights. This modification is beneficial in addressing class imbalances and emphasizing specific class pairings over others.

The function processes predictions $y_{\mathrm{pred}}$ and true labels $y_{\mathrm{true}}$ in a multistep approach, starting with a softmax transformation and followed by the application of class-specific weights in the gradient and Hessian computation. See (Algorithm 6).
**Algorithm 6** Weighted Softmax Loss Function—Variant 1**Require:** *y*, $\hat{y}$$\hat{y}\leftarrow \exp\left(\hat{y}-\max\left(\hat{y}\right)\right)$$\hat{y}\leftarrow \hat{y}/\sum \left(\hat{y}\right)$$grad\leftarrow \mathrm{zero}\;\mathrm{matrix}\;\mathrm{with}\;\mathrm{shape}\;\mathrm{of}\;\hat{y}$$hess\leftarrow \mathrm{zero}\;\mathrm{matrix}\;\mathrm{with}\;\mathrm{shape}\;\mathrm{of}\;\hat{y}$weights←{(0,1):0.1,(1,0):0.1,(0,2):0.17,(2,0):0.17,(1,2):0.1,(2,1):0.1}**for** i=0 to lengthofy−1 **do**    **for** j=0 to $\mathrm{number}\;\mathrm{of}\;\mathrm{columns}\;\mathrm{in}\;\hat{y}-1$ **do**        weight←weights[min(y[i],j),max(y[i],j)]        **if** weight is not set **then**           weight←0        **end if**        $prob\leftarrow \hat{y}\left[i,j\right]$        grad[i,j]←weight×(prob−(y[i]==j))        hess[i,j]←weight×prob×(1−prob)+0.02    **end for****end forreturn**  grad,hess

#### 2.7.1. Softmax Transformation

The softmax transformation is defined as [69]:

(6) $$\hat{y}=\frac{\exp(\hat{y}-\max\left(\hat{y}\right)}{\sum \exp(\hat{y}-\max\left(\hat{y}\right))}$$

#### 2.7.2. Gradient and Hessian Initialization

Both gradient grad and Hessian hess matrices are initialized as zero matrices of the same dimensions as $y_{\mathrm{pred}}$.

#### 2.7.3. Weight Assignment

The function utilizes a predefined dictionary of weights for class pairs. The weights for some of the class pairings are as follows:

- Classes 0 and 1: $w_{0,1}=w_{1,0}=0.1$;
- Classes 0 and 2: $w_{0,2}=w_{2,0}=0.17$;
- Classes 1 and 2: $w_{1,2}=w_{2,1}=0.1$.

These weights are used to scale the impact of each class pair differently, allowing for a customized response to the imbalance or specific importance of class combinations.

#### 2.7.4. Gradient and Hessian Computation

For each instance and class, the gradient and Hessian are computed as follows [70]:

(7) $${\mathrm{grad}}_{ij}=w_{ij}\cdot \left(p_{ij}-⊮\left(y_{i}==j\right)\right)$$

(8) $${\mathrm{hess}}_{ij}=w_{ij}\cdot p_{ij}\cdot \left(1-p_{ij}\right)+\lambda$$

where $p_{ij}$ is the predicted probability of instance *i* belonging to class *j*, ⊮ is the indicator function, and λ is a regularization term to prevent overfitting.

By incorporating class-specific weights, the ‘WeightedSoftmaxLossVariant1’ function provides a nuanced approach to handling classification tasks. This functionality is particularly advantageous in datasets where certain class combinations are more critical or where there is a significant class imbalance. The flexibility to adjust weights as needed allows the function to be tailored to the specific requirements of various datasets and classification challenges.

### 2.8. Weighted Softmax Loss Function—Variant 2

The ‘WeightedSoftmaxLossVariant2’ function is a modification of the earlier described ‘WeightedSoftmaxLossVariant1’. This variant introduces a slight change in the weights assigned to class pairs, refining the approach towards class-specific penalization in classification tasks.

The methodological steps in ‘WeightedSoftmaxLossVariant2’ remain largely the same as in Variant 1, with the primary difference lying in the weight assignment for class pairs.

#### Modified Weight Assignment

The key modification in Variant 2 is in the weight assignment. The new weights for the class pairs are as follows:

- Classes 0 and 1: $w_{0,1}=w_{1,0}=0.1$;
- Classes 0 and 2: $w_{0,2}=w_{2,0}=0.18$;
- Classes 1 and 2: $w_{1,2}=w_{2,1}=0.1$.

The adjustment in weights, particularly for the class pairs (0, 2) and (2, 0), signifies a refined approach to how the function penalizes or prioritizes different class pairs.

The ‘WeightedSoftmaxLossVariant2’ function demonstrates how subtle changes in the weight configuration can lead to significant differences in the behavior of loss functions. By adjusting the weights for specific class pairs, the function becomes more adaptable to the nuances of different classification problems, particularly where specific class pairs require more attention due to imbalance or other specific considerations.

### 2.9. Weighted Softmax Loss Function—Variant 3

The ‘WeightedSoftmaxLossVariant3’ function introduces another iteration of refinements in the Weighted Softmax Loss approach. Similar to its predecessors, this variant adjusts the weights assigned to specific class pairs, refining the model’s sensitivity to different class interactions.

The fundamental methodology of ‘WeightedSoftmaxLossVariant3’ remains consistent with the previous versions, with the primary distinction being the further adjustment of the class pair weights.

#### Modified Weight Assignment

The new weights in ‘WeightedSoftmaxLossVariant3’ are as follows:

- Classes 0 and 1: $w_{0,1}=w_{1,0}=0.1$;
- Classes 0 and 2: $w_{0,2}=w_{2,0}=0.19$;
- Classes 1 and 2: $w_{1,2}=w_{2,1}=0.1$.

This modification, particularly for the class pairs (0, 2) and (2, 0), indicates a continued refinement in the model’s approach to handling these class interactions.

‘WeightedSoftmaxLossVariant3’ further exemplifies the adaptive nature of weighted loss functions in machine learning classification tasks. By incrementally adjusting the weights for specific class pairs, this function allows for a more fine-tuned response to class imbalances and specific classification dynamics. Such iterative refinements highlight the importance of customizing loss functions to suit the specific needs of diverse datasets and classification challenges.

### 2.10. Weighted Softmax Loss Function—Variant 4

The ‘WeightedSoftmaxLossVariant4’ represents the penultimate iteration in the series of modifications to the Weighted Softmax Loss function. This version continues to adjust the weighting strategy for class pairs, reflecting an ongoing refinement to address the specific nuances of classification tasks better.

The overall structure and methodology of ‘WeightedSoftmaxLossVariant4’ remain consistent with its predecessors, with the critical difference being the updated weight values for certain class pairs.

#### Modified Weight Assignment

In ‘WeightedSoftmaxLossVariant4’, the weights for class pairs are adjusted as follows:

- Classes 0 and 1: $w_{0,1}=w_{1,0}=0.1$;
- Classes 0 and 2: $w_{0,2}=w_{2,0}=0.20$;
- Classes 1 and 2: $w_{1,2}=w_{2,1}=0.1$.

This change, especially for the class pairs (0, 2) and (2, 0), indicates a continual refinement in the approach toward handling these specific class interactions.

The progression to ‘WeightedSoftmaxLossVariant4’ underscores the importance of continuous optimization in loss function design, particularly in the context of class imbalances and specific classification dynamics. By fine-tuning the weights assigned to different class pairs, this variant offers an even more nuanced approach to addressing the complexities inherent in diverse datasets and classification challenges.

### 2.11. Weighted Softmax Loss Function—Variant 5

‘WeightedSoftmaxLossVariant5’ represents the culmination of a series of refinements in the Weighted Softmax Loss function series [71]. This final variant further fine-tunes the weighting system for class pairs, aiming to optimize the classification performance in machine learning models.

The methodology for ‘WeightedSoftmaxLossVariant5’ follows the established pattern of its predecessors, with modifications confined to the weights assigned to the class pairs.

#### Modified Weight Assignment

The weights for class pairs in ‘WeightedSoftmaxLossVariant5’ are adjusted as follows:

- Classes 0 and 1: $w_{0,1}=w_{1,0}=0.1$
- Classes 0 and 2: $w_{0,2}=w_{2,0}=0.21$
- Classes 1 and 2: $w_{1,2}=w_{2,1}=0.1$

These adjustments, especially for the class pairs (0, 2) and (2, 0), reflect an ongoing effort to optimize the model’s sensitivity and response to specific class dynamics.

With ‘WeightedSoftmaxLossVariant5’, the series of modifications to the Weighted Softmax Loss function reaches a refined state, demonstrating a meticulous approach to enhancing classification models. By incrementally adjusting the weights for class pairs, this variant offers a sophisticated balance, addressing class imbalances and specific classification challenges with heightened precision. This iterative process highlights the value of continual optimization in loss function design, catering to the complex needs of diverse and evolving datasets.

### 2.12. Weighted Softmax Loss Function with Edge Penalty

The ‘WeightedSoftmaxLossWithEdgePenalty’ function introduces an advanced modification to the standard Weighted Softmax Loss function by incorporating an extra penalty for edge class errors. This enhancement aims to address the challenges posed by class imbalances, particularly when errors in extreme classes (or edge classes) have a more significant impact.

The function maintains the core structure of the Weighted Softmax Loss but introduces an additional penalty factor for errors involving the edge classes.

#### 2.12.1. Modified Weight Assignment

The function assigns weights to class pairs and introduces an extra penalty for errors involving the edge classes.

The weights for class pairs in ‘WeightedSoftmaxLossWithEdgePenalty’ are adjusted as follows:

- Classes 0 and 1: $w_{0,1}=w_{1,0}=0.1$;
- Classes 0 and 2: $w_{0,2}=w_{2,0}=0.17$;
- Classes 1 and 2: $w_{1,2}=w_{2,1}=0.1$.

#### 2.12.2. Algorithm of Weighted Softmax Loss Function with Edge Penalty

The algorithm for the ‘WeightedSoftmaxLossWithEdgePenalty’ function is presented as follows (Algorithm 7).
**Algorithm 7** Weighted Softmax Loss with Edge Penalty**Require:** $y,\hat{y}$Initialize grad and hess as zero matrices of the same shape as $\hat{y}$Define class pair weightsSet extra_penalty←1.2**for** i=0 to len(y)−1 **do**    **for** j=0 to $columns(\hat{y})-1$ **do**        weight← weight for the pair (min(y[i],j),max(y[i],j))        $prob\leftarrow \hat{y}\left[i,j\right]$        penalty←extra_penalty if y[i] or *j* is an edge class, else 1        grad[i,j]←penalty×weight×(prob−(y[i]==j))        hess[i,j]←penalty×weight×prob×(1−prob)+0.02    **end for****end forreturn** grad,hess

The ‘WeightedSoftmaxLossWithEdgePenalty’ function represents a significant advancement in addressing class imbalance and the specific challenges posed by edge classes in classification tasks. By applying an extra penalty to errors involving these classes, the function aims to improve model sensitivity and accuracy in scenarios where edge class errors are particularly costly or significant.

### 2.13. Adaptive Weighted Softmax Loss Function

The Adaptive Weighted Softmax Loss function introduces a dynamic approach to weight assignment in the context of classification errors, combined with a focal loss modification [72] to further enhance model performance, especially in scenarios with imbalanced classes or hard-to-classify instances.

This function comprises two significant components: the computation of adaptive weights based on class-specific errors and the incorporation of the focal loss concept into the softmax loss calculation.

#### 2.13.1. Computing Adaptive Weights

Adaptive weights [73] are computed based on the average error of each class. An additional focus is given to specified class pairs with a focus multiplier. In this implementation, the focus_pairs are set to [(0, 2), (2, 0)], indicating a heightened emphasis on the errors between these class pairs. The focus_multiplier is set to 5.0, significantly amplifying the weight adjustment for these pairs.

#### 2.13.2. Focal Loss Modification

The focal loss [74,75] modification adjusts the contribution of each sample to the loss based on the correctness of its classification, thereby focusing more on difficult or misclassified samples.

#### 2.13.3. Algorithm of Adaptive Weighted Softmax Loss Function

The pseudocode for computing adaptive weights is presented in Algorithm 8, and the Adaptive Weighted Softmax Loss Function is outlined in Algorithm 9.
**Algorithm 8** Compute Adaptive Weights**Require:** $y,\hat{y},focus\_pairs,focus\_multiplier$$n\_classes\leftarrow columns(\hat{y})$Initialize class_errors as a zero vector of length n_classes**for** i=0 to n_classes−1 **do**    class_indices← indices where y=i    class_errors[i]← mean absolute error of $\hat{y}\left[class\_indices,i\right]$ from 1**end for**Normalize class_errors by its maximum valueInitialize weights as an empty dictionary**for** i=0 to n_classes−1 **do**    **for** j=i to n_classes−1 **do**        **if** i=j **then**           weights[(i,j)]←0.1+0.1×class_errors[i]        **else**           avg_error←(class_errors[i]+class_errors[j])/2           weights[(i,j)]←weights[(j,i)]←avg_error           **if** (i,j) in focus_pairs or (j,i) in focus_pairs **then**               weights[(i,j)]←weights[(j,i)]←avg_error×focus_multiplier           **end if**        **end if**    **end for****end forreturn** weights

**Algorithm 9** Adaptive Weighted Softmax Loss with Focal Modification
**Require:** $y,\hat{y},gamma=2$Compute $\hat{y}$ using the softmax transformationInitialize grad and hess as zero matrices of the same shape as $\hat{y}$Compute adaptive weights using *y* and $\hat{y}$**for** i=0 to len(y)−1 **do**    **for** j=0 to $columns(\hat{y})-1$ **do**        weight← weight for the pair (min(y[i],j),max(y[i],j))        $prob\leftarrow \hat{y}\left[i,j\right]$        $focal\_mod\leftarrow {\left(1-prob\right)}^{g}amma$        grad[i,j]←focal_mod×weight×(prob−(y[i]==j))        hess[i,j]←focal_mod×weight×prob×(1−prob)+0.02    **end for****end forreturn** grad,hess


The Adaptive Weighted Softmax Loss function with Focal Modification offers a sophisticated approach to handling complex classification scenarios. By dynamically adjusting weights according to class-specific errors and incorporating the focal loss mechanism, this function aims to improve model accuracy and robustness, particularly in cases of class imbalance or where certain classes are more challenging to classify correctly.

## 3. Numerical Experiments

All features obtained through the five extraction methods were integrated into a single dataset. However, a distinction was made to identify the drill to which each feature set belongs. This design choice was critical for maintaining the integrity of the data and ensuring that the classification model accurately reflects the characteristics specific to each drill.

In our experiments, we opted not to apply feature selection for the XGBoost classifier. This decision was grounded in several considerations specific to the nature of our data and the inherent characteristics of XGBoost. Here are the key reasons for this approach:

- Inherent Feature Handling Capabilities of XGBoost: XGBoost is well-known for its ability to handle a large number of features efficiently. It automatically assigns a score to each feature based on its importance, effectively doing an internal form of feature selection during the learning process. Given this capability, we believed that an additional explicit feature selection step might not significantly improve the performance.
- Complexity of the Data: The dataset in our study was complex, with features extracted from five different methods. Each feature potentially carried unique information that could be crucial for accurate classification. We wanted to ensure that the model had access to all available information before making any decision to exclude features.
- Avoiding Potential Loss of Information: Feature selection, especially if not done carefully, can lead to the loss of important information that could be valuable for the model. Given the critical nature of our task—drill-wear analysis—we could not afford to lose potentially subtle yet important signals that might be present in the less prominent features.
- Computational Resources: We had access to sufficient computational resources to handle the complexity and size of our dataset without the need for feature reduction. This allowed us to train the XGBoost model on the full set of features without concerns about computational efficiency or training time.
- Ensuring Model Robustness: By using the complete set of features, we aimed to develop a model that is robust and can generalize well across different scenarios. Reducing the feature space might lead to a model that is overly optimized for the specific characteristics of the training data, potentially reducing its effectiveness on new, unseen data.

Thus, considering the strengths of XGBoost in handling high-dimensional data, along with the desire to preserve the integrity and richness of our dataset, we concluded that feature selection was not a necessary preprocessing step for our specific application. The results of our experiments would provide insights into the effectiveness of this approach.

In the course of this study, a conscious decision was made to not engage in hyperparameter optimization. This approach was grounded in our intent to demonstrate that modifications to the loss function alone can optimize the classifier for the specific problem at hand. Hyperparameter optimization, by its nature, is not deterministic and often involves elements of randomness. In our methodology, we sought to ensure that the comparison of different loss function methods was as reliable and consistent as possible. Therefore, to maintain stability and determinism in our experiments, elements prone to nondeterminism and randomness, such as feature selection and hyperparameter optimization, were deliberately omitted. This approach allowed us to focus on the direct impact of the loss function modifications, ensuring that any observed improvements in classifier performance could be attributed specifically to these changes rather than to variations in other parameters of the model.

Industry experts who designed the entire experimental setup imposed a specific process for validation (cross-validation). The experiment involved five drills, and a five-fold cross-validation was applied. However, in this particular experiment, a ‘fold’ corresponds to one drill. This means that in each step of the validation process, one drill is used as the test set, while data from the remaining four drills are utilized for training the model. This procedure is repeated five times, once for each drill.

Industry experts mandated this five-stage cross-validation process. It was specifically chosen to ensure that the model’s performance is robust and generalizable across different drills. This approach also addresses potential biases that might arise if the model is trained and tested on data from the same drill. By separating the test and training sets based on the drill, the validation process provides a more reliable assessment of the model’s effectiveness and accuracy in real-world conditions.

Additionally, it is essential to note that the custom loss functions were implemented using Python 3.9. For the classifier, the implementation of XGBoost used was DLMC XGBoost [76]. This library is an optimized distributed Gradient Boosting code renowned for its efficiency, flexibility, and portability. It implements machine learning algorithms under the Gradient Boosting framework, providing a parallel tree boosting technique, also known as GBDT (Gradient Boosting Decision Tree) or GBM (Gradient Boosting Machine). The use of DLMC XGBoost in our experiments ensures that our model not only leverages a state-of-the-art algorithm for boosted tree learning but also benefits from the latest advancements in distributed computing for large-scale machine learning tasks.

All experiments were performed on hardware (Ubuntu operating system) with the following specifications:

- Processor: AMD RYZEN THREADRIPPER 2990WX (32C 64T) 4.3 GHz;
- Motherboard: AsRock X399 TAICHI;
- Memory: 8 × ADATA XPG SPECTRIX DDR4 16 GB D41 3000 MHz (128 GB RAM);
- Graphics Card: 2 × Nvidia GeForce RTX Titan 24 GB GDDR6 (48 GB RAM);
- Drive SSD: 2 × WD BLACK 1 TB WDS100T3X0C1TB (PCIE);
- Drive HDD: 1 × WD RED PRO 8 TB WD8003FFBX 3.5” (SATA);
- Power Supply: BE QUIET! DARK POWER PRO 11 1000 W;
- Cooling: BE QUIET! Silent Loop BW003 280 mm;
- Network: 10GbE SFP+.

## 4. Results and Discussion

The experimental evaluation of various XGBoost loss functions, with a particular focus on the reduction in critical errors in classification tasks, has been presented below. Table 3 presents a comprehensive comparative analysis of these loss functions.

The Default Softmax Loss Function, serving as a baseline, showed a total of 1150 critical errors (552 Green-Red errors and 598 Red-Green errors), with an overall accuracy of 64.29%. This standard metric sets the stage for evaluating the enhancements achieved through the implementation of weighted loss functions.

A noticeable trend in the data is the gradual reduction in the total count of critical errors as we progress from Weighted Softmax Loss Variant 1 to Variant 5. Notably, Variant 1 exhibited a slightly higher Green-Red error count (683) compared to the Default Softmax Loss but a reduced Red-Green error count (457), leading to a marginally decreased overall accuracy of 62.73%.

Variants 2 through 5 consistently showed a decrease in both Green-Red and Red-Green errors, culminating in Variant 5, which recorded the lowest total critical errors (853) among these variants. Despite this improvement, there was a noticeable decrease in overall accuracy, with Variant 5 reaching 59.73%.

The Weighted Softmax Loss with Edge Penalty and the Adaptive Weighted Softmax Loss introduced more nuanced approaches to handling critical errors. The former resulted in a total of 873 critical errors and an accuracy of 60.59%, while the latter achieved the most substantial reduction in critical errors, totaling 724 with an accuracy of 56.08%.

**Figure 5:**
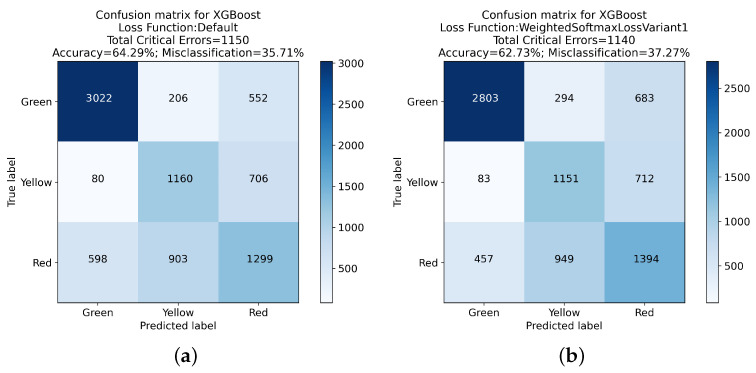
Confusion matrix analysis for XGBoost with Default Loss Function and Variant 1: (**a**) presents the classification outcomes using the Default Loss Function, while (**b**) illustrates results from Weighted Softmax Loss Function Variant 1.

**Figure 6:**
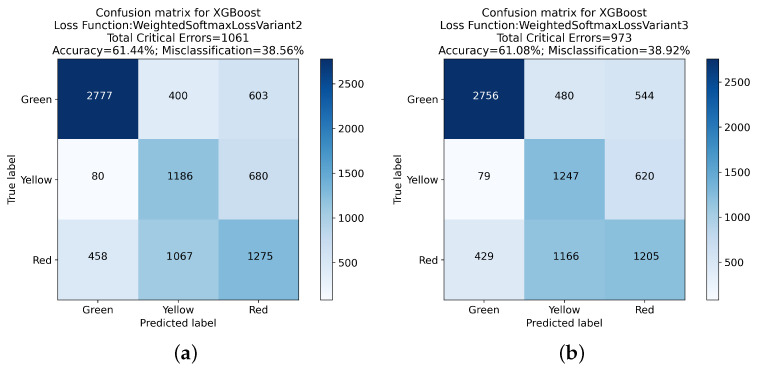
Confusion matrix analysis for XGBoost with Weighted Softmax Loss Function Variant 2 and Variant 3: (**a**) presents the classification outcomes using the Weighted Softmax Loss Function Variant 2, while (**b**) illustrates results from Weighted Softmax Loss Function Variant 3.

**Figure 7:**
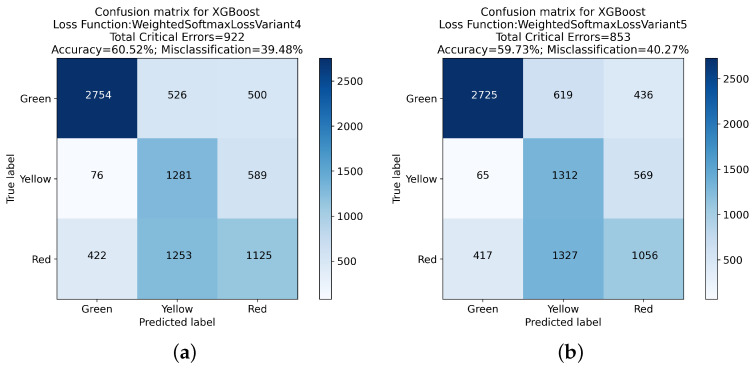
Confusion matrix analysis for XGBoost with Weighted Softmax Loss Variant 4 and Variant 5: (**a**) presents the classification outcomes using the Weighted Softmax Loss Variant 4, while (**b**) illustrates results from Weighted Softmax Loss Variant 5.

**Figure 8:**
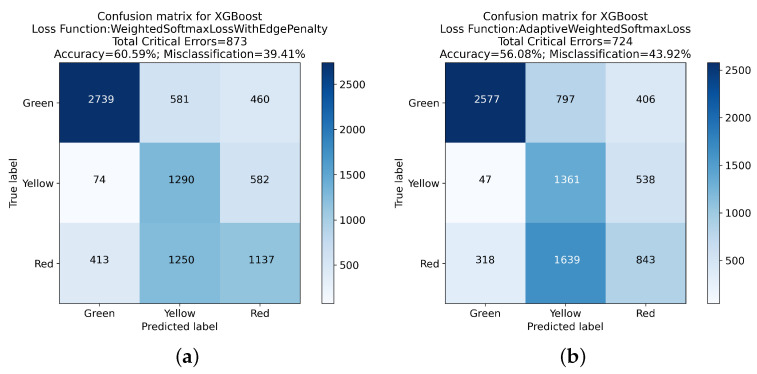
Confusion matrix analysis for XGBoost with Weighted Softmax Loss Function with Edge Penalty and Adaptive Weighted Softmax Loss Function: (**a**) presents the classification outcomes using the Weighted Softmax Loss Function with Edge Penalty, while (**b**) illustrates results from Adaptive Weighted Softmax Loss Function.

These findings are further elucidated in the confusion matrices presented in Figure 5, Figure 6, Figure 7 and Figure 8. These matrices provide a detailed view of the classification performance across different classes, offering a visual representation of the trade-offs between specific error types and overall accuracy.

The exploration of different loss functions in the XGBoost algorithm reveals a complex balance between reducing critical errors and maintaining overall classification accuracy. The insights gained from this analysis, mainly when considered alongside the detailed results in the confusion matrices, are invaluable for guiding the choice of loss function in practical applications, depending on the specific requirements of the classification task at hand.

Another crucial aspect to consider is the computational time, which reflects the efficiency and practical applicability of these algorithms in real-world scenarios.

Looking at the “Time” column in Table 3, we note a consistent trend in the computational time required for each variant of the loss function. The Default Softmax Loss function, serving as our baseline, required 199 s. As we progressed through the Weighted Softmax Loss Variants 1 through 5, there was a slight but consistent increase in computation time, culminating in Variant 5, which took 218 s. This increment can be attributed to the additional complexity introduced by the weighted mechanism in these variants.

The Weighted Softmax Loss with Edge Penalty and the Adaptive Weighted Softmax Loss Function exhibited more significant increases in computational time, 232 s and 473 s, respectively. The additional time for the Edge Penalty variant might be due to the extra computations required for applying penalties to the edge classes. However, the Adaptive Weighted Softmax Loss Function, which showed the most substantial reduction in critical errors, also required the longest computational time. This increase in time is likely due to the dynamic nature of the adaptive weights calculation and the incorporation of the focal loss mechanism, which adds to the computational complexity.

Below, a detailed analysis of the performance metrics for different loss functions applied to the XGBoost algorithm is presented. These metrics include precision, sensitivity (recall), F1 score, and specificity, evaluated for each class (Green, Yellow, and Red).

The detailed analysis presented in Table 4, Table 5, Table 6, Table 7, Table 8, Table 9, Table 10 and Table 11 plays a crucial role in understanding the nuances of our study. These tables collectively provide a comprehensive evaluation of the performance metrics for various loss functions applied in the XGBoost algorithm, offering insights into their effectiveness in the context of drill-wear analysis for melamine-faced chipboard.

Table 4 presents the baseline performance metrics using the Default Softmax Loss Function. This sets the stage for comparison with other loss function variants. It is observed that while the Green class shows relatively high precision and sensitivity, the Yellow and Red classes have lower values, indicating potential areas for improvement.

Table 5, Table 6, Table 7, Table 8 and Table 9 depict the results for Weighted Softmax Loss Function Variants 1 through 5. A noticeable trend across these variants is the increasing precision for the Green class but with a decrease in sensitivity, suggesting a more conservative classification. The Yellow and Red classes, however, show mixed results, indicating the complexities involved in balancing precision and recall for these classes.

In Table 10, we observe the performance metrics for the Weighted Softmax Loss Function with Edge Penalty. This variant shows a significant improvement in precision and recall for the Yellow class, suggesting a more balanced classification across the classes. However, the Red class continues to be challenging in terms of lower precision and recall.

Lastly, Table 11 focuses on the Adaptive Weighted Softmax Loss Function. While this variant demonstrates a notable shift in performance with a significant improvement for the Yellow class, it highlights the ongoing challenge with the Red class, which exhibits the lowest precision and recall.

The discussions surrounding these tables emphasize the trade-offs inherent in optimizing loss functions for multi-class classification tasks. The variation in performance metrics across different classes underscores the complexity of achieving a balance between precision and recall and highlights the importance of selecting a loss function that aligns with the specific needs and objectives of the task at hand.

In conclusion, the exploration of different XGBoost loss functions reveals significant variations in performance metrics across different classes. While some loss functions improve precision or recall for certain classes, they often do so at the expense of other metrics or classes. This underscores the challenge of finding a one-size-fits-all loss function for multi-class classification tasks and highlights the importance of choosing a loss function that aligns with specific objectives and class priorities of the given task.

### 4.1. Advantages and Limitations of the Proposed Work

#### 4.1.1. Advantages

The proposed approach of applying custom loss functions in XGBoost for drill-wear analysis in melamine-faced chipboard presents several significant advantages:

- Enhanced Accuracy for Critical Classes: By customizing loss functions, the model demonstrates improved classification accuracy, especially for critical edge classes, which are essential in industrial applications for maintaining production quality.
- Flexibility in Addressing Class Imbalance: The adaptive nature of the proposed loss functions effectively addresses the challenges posed by imbalanced datasets, a common issue in real-world scenarios.
- Context-Specific Model Optimization: Tailoring loss functions according to the specific needs of the application allows for a more nuanced and effective model compared to standard approaches.
- Improved Decision-Making in Industrial Settings: The refined predictions offered by the model facilitate better decision-making processes, crucial in high-stakes industrial environments such as furniture manufacturing.

#### 4.1.2. Limitations

Despite its advantages, the proposed approach also exhibits certain limitations that should be considered:

- Increased Computational Complexity: Custom loss functions, particularly those involving adaptive weights and focal modifications, demand higher computational resources, potentially impacting the efficiency of the model.
- Overfitting Risks: The model’s heightened sensitivity to specific classes might lead to overfitting, particularly when dealing with small or highly specific datasets.
- Dependency on Expert Knowledge: The effectiveness of the approach relies heavily on domain expertise for accurately defining and tuning the custom loss functions.
- Limited Generalizability: While effective in the specific context of drill-wear analysis, the approach may not be directly applicable or as effective in other domains without significant modifications.

## 5. Conclusions

This study advances the understanding of custom loss functions in the XGBoost algorithm, specifically tailored for the complex task of drill-wear analysis in melamine-faced chipboard processing. The exploration reveals that the Adaptive Weighted Softmax Loss Function markedly reduces critical errors in multi-class classification, setting a new benchmark compared to the baseline Default Softmax Loss Function. The investigation into various Weighted Softmax Loss Function variants illustrates a nuanced trade-off between minimizing critical errors and maintaining overall classification accuracy. This highlights the complexity and intricacies involved in optimizing these aspects simultaneously.

While the Adaptive Weighted Softmax Loss Function demonstrates superior performance in reducing critical errors, it comes at the cost of increased computational time. This trade-off between performance and efficiency is a critical consideration in practical applications. For scenarios where time efficiency is a priority, simpler models such as the Default Softmax or the earlier variants of the Weighted Softmax Loss might be preferable. However, for applications where the reduction in critical errors is paramount and computational resources are not a constraint, the Adaptive Weighted Softmax Loss Function presents an effective solution despite its higher computational demand.

Furthermore, the research underscores the significance of selecting loss functions that align with the specific challenges and objectives of classification tasks, especially in scenarios with high sensitivity to critical errors. For practitioners in the field of industrial machine learning, this study offers valuable insights into the choice of loss functions based on the specific requirements and priorities of the task at hand.

In summary, the findings of this research contribute significantly to the practical application of machine learning in industrial settings. By demonstrating the effectiveness of custom loss functions in addressing specialized classification challenges, this study paves the way for the development of more accurate and reliable classification models in various industrial applications.

## Figures and Tables

**Table 1:** Breakdown of image counts for each drill class in the dataset.

Drill Number	Green Class	Yellow Class	Red Class	Total
Drill 1	840	420	406	1666
Drill 2	840	700	280	1820
Drill 3	700	560	420	1680
Drill 4	840	560	280	1680
Drill 5	560	560	560	1680
Total	3780	2800	1946	8526

**Table 2:** Detailed layer-by-layer description of the ResNet-18 network architecture. Layers 35 and 68, highlighted in the table, are used for extracting lower-level and high-level features, respectively.

No.	Layer’s Name	Layer’s Type	Description	Total Learnables
1	data	Image Input	224 × 224 × 3 images with ‘zscore’ normalization	0
2	conv1	2-D Convolution	64 7 × 7 × 3 convolutions with stride [2 2] and padding [3 3 3 3]	9472
3	bn_conv1	Batch Normalization	Batch normalization with 64 channels	128
4	conv1_relu	ReLU	ReLU	0
5	pool1	2-D Max Pooling	3 × 3 max pooling with stride [2 2] and padding [1 1 1 1]	0
6	res2a_branch2a	2-D Convolution	64 3 × 3 × 64 convolutions with stride [1 1] and padding [1 1 1 1]	36,928
7	bn2a_branch2a	Batch Normalization	Batch normalization with 64 channels	128
8	res2a_branch2a_relu	ReLU	ReLU	0
9	res2a_branch2b	2-D Convolution	64 3 × 3 × 64 convolutions with stride [1 1] and padding [1 1 1 1]	36,928
10	bn2a_branch2b	Batch Normalization	Batch normalization with 64 channels	128
11	res2a	Addition	Element-wise addition of 2 inputs	0
12	res2a_relu	ReLU	ReLU	0
13	res2b_branch2a	2-D Convolution	64 3 × 3 × 64 convolutions with stride [1 1] and padding [1 1 1 1]	36,928
14	bn2b_branch2a	Batch Normalization	Batch normalization with 64 channels	128
15	res2b_branch2a_relu	ReLU	ReLU	0
16	res2b_branch2b	2-D Convolution	64 3 × 3 × 64 convolutions with stride [1 1] and padding [1 1 1 1]	36,928
17	bn2b_branch2b	Batch Normalization	Batch normalization with 64 channels	128
18	res2b	Addition	Element-wise addition of 2 inputs	0
19	res2b_relu	ReLU	ReLU	0
20	res3a_branch2a	2-D Convolution	128 3 × 3 × 64 convolutions with stride [2 2] and padding [1 1 1 1]	73,856
21	bn3a_branch2a	Batch Normalization	Batch normalization with 128 channels	256
22	res3a_branch2a_relu	ReLU	ReLU	0
23	res3a_branch2b	2-D Convolution	128 3 × 3 × 128 convolutions with stride [1 1] and padding [1 1 1 1]	147,584
24	bn3a_branch2b	Batch Normalization	Batch normalization with 128 channels	256
25	res3a_branch1	2-D Convolution	128 1 × 1 × 64 convolutions with stride [2 2] and padding [0 0 0 0]	8320
26	bn3a_branch1	Batch Normalization	Batch normalization with 128 channels	256
27	res3a	Addition	Element-wise addition of 2 inputs	0
28	res3a_relu	ReLU	ReLU	0
29	res3b_branch2a	2-D Convolution	128 3 × 3 × 128 convolutions with stride [1 1] and padding [1 1 1 1]	147,584
30	bn3b_branch2a	Batch Normalization	Batch normalization with 128 channels	256
31	res3b_branch2a_relu	ReLU	ReLU	0
32	res3b_branch2b	2-D Convolution	128 3 × 3 × 128 convolutions with stride [1 1] and padding [1 1 1 1]	147,584
33	bn3b_branch2b	Batch Normalization	Batch normalization with 128 channels	256
34	res3b	Addition	Element-wise addition of 2 inputs	0
35	res3b_relu	ReLU	ReLU	0
36	res4a_branch2a	2-D Convolution	256 3 × 3 × 128 convolutions with stride [2 2] and padding [1 1 1 1]	295,168
37	bn4a_branch2a	Batch Normalization	Batch normalization with 256 channels	512
38	res4a_branch2a_relu	ReLU	ReLU	0
39	res4a_branch2b	2-D Convolution	256 3 × 3 × 256 convolutions with stride [1 1] and padding [1 1 1 1]	590,080
40	bn4a_branch2b	Batch Normalization	Batch normalization with 256 channels	512
41	res4a_branch1	2-D Convolution	256 1 × 1 × 128 convolutions with stride [2 2] and padding [0 0 0 0]	33,024
42	bn4a_branch1	Batch Normalization	Batch normalization with 256 channels	512
43	res4a	Addition	Element-wise addition of 2 inputs	0
44	res4a_relu	ReLU	ReLU	0
45	res4b_branch2a	2-D Convolution	256 3 × 3 × 256 convolutions with stride [1 1] and padding [1 1 1 1]	590,080
46	bn4b_branch2a	Batch Normalization	Batch normalization with 256 channels	512
47	res4b_branch2a_relu	ReLU	ReLU	0
48	res4b_branch2b	2-D Convolution	256 3 × 3 × 256 convolutions with stride [1 1] and padding [1 1 1 1]	590,080
49	bn4b_branch2b	Batch Normalization	Batch normalization with 256 channels	512
50	res4b	Addition	Element-wise addition of 2 inputs	0
51	res4b_relu	ReLU	ReLU	0
52	res5a_branch2a	2-D Convolution	512 3 × 3 × 256 convolutions with stride [2 2] and padding [1 1 1 1]	1,180,160
53	bn5a_branch2a	Batch Normalization	Batch normalization with 512 channels	1024
54	res5a_branch2a_relu	ReLU	ReLU	0
55	res5a_branch2b	2-D Convolution	512 3 × 3 × 512 convolutions with stride [1 1] and padding [1 1 1 1]	2,359,808
56	bn5a_branch2b	Batch Normalization	Batch normalization with 512 channels	1024
57	res5a_branch1	2-D Convolution	512 1 × 1 × 256 convolutions with stride [2 2] and padding [0 0 0 0]	131,584
58	bn5a_branch1	Batch Normalization	Batch normalization with 512 channels	1024
59	res5a	Addition	Element-wise addition of 2 inputs	0
60	res5a_relu	ReLU	ReLU	0
61	res5b_branch2a	2-D Convolution	512 3 × 3 × 512 convolutions with stride [1 1] and padding [1 1 1 1]	2,359,808
62	bn5b_branch2a	Batch Normalization	Batch normalization with 512 channels	1024
63	res5b_branch2a_relu	ReLU	ReLU	0
64	res5b_branch2b	2-D Convolution	512 3 × 3 × 512 convolutions with stride [1 1] and padding [1 1 1 1]	2,359,808
65	bn5b_branch2b	Batch Normalization	Batch normalization with 512 channels	1024
66	res5b	Addition	Element-wise addition of 2 inputs	0
67	res5b_relu	ReLU	ReLU	0
68	pool5	2-D Global Average Pooling	2-D global average pooling	0
69	fc1000	Fully Connected	1000 fully connected layer	513,000
70	prob	Softmax	softmax	0
71	ClassificationLayer _predictions	Classification Output	crossentropyex with ‘tench’ and 999 other classes	0

**Table 3:** Comparative analysis of different XGBoost loss functions focusing on the reduction in critical errors.

Loss Function for XGBoost	Green-Red Error	Red-Green Error	Total Critical Errors	Accuracy	Time
Default Softmax Loss	552	598	1150	64.29%	199 s
Weighted Softmax Loss V1	683	457	1140	62.73%	215 s
Weighted Softmax Loss V2	603	458	1061	61.44%	214 s
Weighted Softmax Loss V3	544	429	973	61.08%	217 s
Weighted Softmax Loss V4	500	422	922	60.52%	223 s
Weighted Softmax Loss V5	436	417	853	59.73%	218 s
Weighted Softmax Loss With Edge Penalty	460	413	873	60.59%	232 s
Adaptive Weighted Softmax Loss	406	318	724	56.08%	473 s

**Table 4:** Performance metrics for XGBoost using Default Softmax Loss Function.

Class	Precision	Sensitivity	F1 Score	Specificity
Green	81.68%	79.95%	80.80%	85.71%
Yellow	51.12%	59.61%	55.04%	83.15%
Red	50.80%	46.39%	48.50%	78.03%

**Table 5:** Performance metrics for XGBoost using Weighted Softmax Loss Function Variant 1.

Class	Precision	Sensitivity	F1 Score	Specificity
Green	83.85%	74.15%	78.70%	88.62%
Yellow	48.08%	59.15%	53.04%	81.11%
Red	49.98%	49.79%	49.88%	75.64%

**Table 6:** Performance metrics for XGBoost using Weighted Softmax Loss Function Variant 2.

Class	Precision	Sensitivity	F1 Score	Specificity
Green	83.77%	73.47%	78.28%	88.66%
Yellow	44.70%	60.95%	51.58%	77.71%
Red	49.84%	45.54%	47.59%	77.59%

**Table 7:** Performance metrics for XGBoost using Weighted Softmax Loss Function Variant 3.

Class	Precision	Sensitivity	F1 Score	Specificity
Green	84.44%	72.91%	78.25%	89.30%
Yellow	43.10%	64.08%	51.54%	74.98%
Red	50.87%	43.04%	46.62%	79.67%

**Table 8:** Performance metrics for XGBoost using Weighted Softmax Loss Function Variant 4.

Class	Precision	Sensitivity	F1 Score	Specificity
Green	84.69%	72.86%	78.33%	89.51%
Yellow	41.86%	65.83%	51.18%	72.96%
Red	50.81%	40.18%	44.87%	80.98%

**Table 9:** Performance metrics for XGBoost using Weighted Softmax Loss Function Variant 5.

Class	Precision	Sensitivity	F1 Score	Specificity
Green	84.97%	72.09%	78.00%	89.84%
Yellow	40.27%	67.42%	50.42%	70.43%
Red	51.24%	37.71%	43.45%	82.45%

**Table 10:** Performance metrics for XGBoost using Weighted Softmax Loss Function with Edge Penalty.

Class	Precision	Sensitivity	F1 Score	Specificity
Green	73.37%	72.46%	72.91%	81.08%
Yellow	70.72%	66.29%	68.44%	89.89%
Red	38.30%	40.61%	39.42%	71.88%

**Table 11:** Performance metrics for XGBoost using Adaptive Weighted Softmax Loss Function.

Class	Precision	Sensitivity	F1 Score	Specificity
Green	69.80%	68.17%	68.98%	79.71%
Yellow	75.03%	69.94%	72.39%	90.15%
Red	27.91%	30.11%	28.97%	68.72%

## Data Availability

Dataset available on request from the authors.
